# Risk assessment of two new pesticides based on the intestinal fungal community construction and growth status of predatory insects (*Arma custos*)

**DOI:** 10.3389/fmicb.2025.1686765

**Published:** 2025-11-27

**Authors:** Xue Yang, Fei Xu, Baoshan Zhang, Maojin Huang, Guocai Zhang

**Affiliations:** 1 College of Forestry, Northeast Forestry University, Harbin, China; 2 College of Environmental Science and Engineering, China West Normal University, Nanchong, China

**Keywords:** *Arma custos*, intestinal fungi, tetraniliprole, *Metarhizium anisopliae*, community construction, function prediction

## Abstract

This study aims to conduct a comprehensive safety assessment of two novel pesticides—tetraniliprole (SZ) and *Metarhizium anisopliae* (LJ)—with the goal of providing scientific data to underpin the refinement of current risk assessment frameworks for new pesticides and guiding the optimization of field application strategies. Using the predatory insect *A. custos* as a model organism, we designed negative control (CK) and concentration-gradient experiment to investigate the effects of these pesticides on the physiological indices and growth performance of *A. custos*. Additionally, we analyzed their impacts on the structural and functional characteristics of the intestinal fungal community in *A. custos*, as well as the interaction mechanisms between gut fungi and host physiological-biochemical processes. The results showed that: (1) at 72 h post-pesticide application, the body weight growth rate of *A. custos* was higher in the SZD and SZG groups than in the control (CK), with the order SZD > SZG > CK; similarly, the LJG and LJD groups also exhibited higher growth rates than CK, following LJG > LJD > CK. (2) By 120 h post-application, the survival rate of CK was higher than that of the SZD and SZG groups (CK > SZG > SZD), and also higher than that of the LJG and LJD groups (CK > LJG > LJD). (3) The abundance of *M. anisopliae* was positively correlated with the activities of catalase (CAT), carboxylesterase (CarE), superoxide dismutase (SOD), glutathione S-transferase (GST), and acetylcholinesterase (AChE) in *A. custos* (*p* < 0.05). Conversely, tetraniliprole was negatively correlated with CAT and CarE activities (*p* < 0.05). (4) Deterministic ecological processes were the dominant drivers shaping the assembly of the intestinal fungal community in *A. custos* under the LJ and SZ treatments. (5) In the LJ treatment group, *M. anisopliae* absolutely dominated the intestinal fungal community, suppressing the growth of other fungal taxa. In the SZ group, environmentally widespread species such as *Candida parapsilosis* and *Aspergillus penicillioides* became characteristic intestinal fungal groups. In conclusion, *M. anisopliae* showed higher safety for predatory insects compared to tetraniliprole. These findings facilitate the construction of more robust risk assessment frameworks for novel pesticides and provide theoretical insights for promoting sustainable agricultural practices within the ecological civilization development paradigm.

## 1 Introduction

Predatory insects serve as vital ecological regulators, playing an indispensable role in maintaining ecological balance, controlling harmful organism populations, and safeguarding biodiversity (Wang et al., 2022; Bouvet et al., 2019; Rathnayake et al., 2019). However, global population growth and escalating food demands have intensified the issue of excessive pesticide application (Yang Y. et al., 2024; Duque et al., 2024). This practice has triggered mass mortality of non-target predatory insects, severely threatening ecosystem equilibrium and destabilizing natural community dynamics (Kaur et al., 2024). Studies have shown that sublethal exposure to common chemical insecticides (e.g., spinetoram) disrupts survival dynamics and predatory efficiency in *Orius strigicollis*. Long-term reliance on such chemicals represents a primary driver of *O. strigicollis* population collapse in loquat orchards (Lin et al., 2024). Similarly, chronic exposure to sublethal concentrations of cycloxaprid—a novel neonicotinoid insecticide—induces population decline in *Coccinella septempunctata*, compromising its pest control services in agricultural ecosystems (Wu et al., 2022). Spirotetramat, widely used against piercing-sucking pests like *Bemisia tabaci*, exhibits multi-generational impacts on natural enemies. Exposure to sublethal doses (LC10, LC30, LC50) causes significant reductions in *Encarsia formosa* survival rates, decreases in *Bemisia tabaci* parasitism efficiency, and prolonged developmental duration in F1 generation individuals (Yang S. W. et al., 2024). These findings highlight cascading ecological risks beyond direct toxicity. Against the backdrop of modern ecological civilization initiatives, insecticide research, development, and application standards must transcend mere pest control efficacy (Gould et al., 2018; Patel et al., 2025). Risk assessment frameworks should prioritize impacts on non-target natural enemy insects as core evaluation criteria, ensuring agricultural practices balance productivity with ecosystem sustainability (Mandal et al., 2024; Calvo-Agudo et al., 2019).

In the development of novel pesticides, tetraniliprole and *Metarhizium anisopliae* have garnered significant attention as archetypal representatives of new chemical pesticides and biological insecticides, respectively (Ullah et al., 2025; Martínez et al., 2022). Each exhibits unique advantages in agricultural pest management: tetraniliprole demonstrates strong systemic activity, high insecticidal efficacy, low mammalian toxicity, and environmental compatibility (Sun et al., 2024; Qu et al., 2024; Arshad and Joseph, 2024). However, this compound poses significant risks to non-target organisms, particularly by exerting pronounced toxicological impacts on invertebrates such as *Habrobracon hebetor*. These effects manifest as inhibitions of critical biological parameters, including net reproductive rates, intrinsic rates of population increase, and age-specific survival rates, thereby disrupting the demographic stability and ecological functionality of these non-target species (Fooladi et al., 2025). Conversely, *M. anisopliae* faces ecological limitations: its efficacy is compromised by extreme environmental conditions (e.g., high-temperature drought or low-temperature humidity), which suppress spore viability and mycelial growth (Aynalem et al., 2022). Additionally, this biopesticide carries inherent risks of non-target toxicity, though its mechanistic basis remains under characterized (Matsui et al., 2022; Khun et al., 2020). Current risk assessment frameworks for these novel pesticides suffer from critical gaps (Walker et al., 2018). Most existing studies focus narrowly on toxicity toward pollinators or economic insects (e.g., silkworms, honeybees), while the impacts on predatory insects—including lady beetles, lacewings, and predatory mites—remain poorly understood (Fan et al., 2023; Zhu et al., 2023). Notably, the interaction mechanisms between biopesticides like *M. anisopliae* and their predatory insect hosts—particularly at the physiological, ecological, and molecular levels—are virtually unexplored (Keppanan et al., 2017). This knowledge deficit hinders comprehensive evaluations of the ecosystem-level risks posed by these compounds (Rosa-Fontana et al., 2025; Benbrook et al., 2021). To address this, there is an urgent need for multi-dimensional investigations integrating toxicology, ecology, and molecular biology (Barrero-Canosa et al., 2025). Such studies will enhance the scientific rigor of ecological risk assessments and underpin the development of sustainable agricultural practices that balance pest control efficacy with biodiversity conservation (Zhao and Chen, 2023; Tarazona et al., 2024).

Against this backdrop, this study employs the predatory insect *A. custos* as a model organism to investigate the effects of tetraniliprole and *M. anisopliae* on its physiological indices and growth performance. Using control (CK) and concentration-gradient experimental designs (Wang et al., 2021; Tosi et al., 2022), we first characterized dose-toxicity responses of growth indicators and enzyme activities of *A. custos* to both pesticides. Building on these experimental foundations, high-throughput sequencing coupled with bioinformatics analyses was used to dissect the regulatory impacts of the pesticides on the structural composition and functional profiles of the gut fungal community in *A. custos*, as well as the interaction mechanisms between gut fungi and host physiological-biochemical pathways. The study aims to: (1) conduct a comprehensive safety assessment of the two pesticides; (2) provide scientific data to underpin the development of risk assessment frameworks for novel pesticides; (3) provide recommendations for eco-friendly field pesticide application; and (4) offer theoretical insights for promoting sustainable agricultural practices within the ecological civilization construction paradigm.

## 2 Materials and methods

### 2.1 Insects

The *A. custos* were provided by the Key Laboratory of Southwest China Wildlife Resource Conservation. These individuals were reared in a growth chamber maintained at optimal temperature and humidity conditions (26 ± 1 °C, 60% ± 5% relative humidity), with a 12:12 h light/dark cycle. They were supplied with fresh water and live Tenebrio molitor as food, while the Tenebrio molitor larvae were fed an aseptic diet to ensure nutritional safety (Cai et al., 2022).

### 2.2 Tetraniliprole

Tetraniliprole was provided by Chengdu Sitiande Biotechnology Co., Ltd. It has a molecular formula of C_22_H_1_6ClF_3_N_10_O_2_, a purity of 97.3%, and should be stored in the dark at −20 °C. The CAS number for this compound is 1229654-66-3.

### 2.3 Entomopathogenic fungi and fungal cultivation

The *M. anisopliae* strain CQMa421 was isolated from field-collected cadavers of *Ceracris kiansu* Tsai and preserved at the China General Microbiological Culture Collection Center (CGMCC) with the registration number CGMCC NO. 1877. Preservation, activation, and expanded cultivation of the experimental strain CQMa421 were performed using methods described by Jiang et al. (2020).

### 2.4 Experimental design of topical dorsal infection with *M. anisopliae* of *Arma custos*

In this experiment, 1,000 fertilized eggs of *A. custos* produced within 12 h were selected for aseptic hatching and cultivation. Following complete hatching and 24 h of development into the 5th instar stage, pesticide treatments were initiated. To characterize *A. custos* sensitivity to the two novel pesticides—*M. anisopliae* and tetraniliprole—pesticides were applied via dual exposure routes: topical application to the insect body and oral administration via treated bait. Treated bait was provided 1 h post-application to allow pesticide absorption. Experimental design followed manufacturer guidelines and preliminary laboratory data, comprising three main groups: *Metarhizium* biopesticide treatment (LJ), tetraniliprole chemical pesticide treatment (SZ), and a negative control (CK). The LJ group was further divided into high (LJG, 1 × 10^8^ conidia⋅mL^−1^) and low (LJD, 1 × 10^5^ conidia⋅mL^−1^) concentration subgroups (Jiang et al., 2020). The SZ group included high (SZG, 200 mg⋅L^−1^) and low (SZD, 20 mg⋅L^−1^) concentration subgroups (Wakil et al., 2025; Ma et al., 2021). The CK group used 0.05% Tween 80 as a vehicle control.

In the experiment for determining the growth and survival rate of *A*. *custos*, five technical replicates were set up for each pesticide-treated group, with 15 *A*. *custos* individuals per technical replicate. Due to the occurrence of intraspecific cannibalism in *A*. *custos*, the 15 individuals in each replicate were reared separately in five insect-rearing bottles (three individuals per bottle). The weight measurement of *A*. *custos* was conducted simultaneously with the mortality recording, and dead individuals were excluded from subsequent weight measurements. In the enzyme activity determination experiment, each pesticide-treated group was also provided with three technical replicates, and each technical replicate (containing 15 *A*. *custos* individuals) was reared in five insect-rearing bottles (three individuals per bottle) following the same method. The total number of *A*. *custos* reared across all groups in the above two experiments was 600. The selected insects met the criteria of uniform body weight, robust development, and high vitality. After pesticide application, the growth and enzyme activity indicators of the insects were monitored once every 24 h (Cheng et al., 2023). At 120 h, three live individuals were randomly sampled from each treatment group, and entire intestines were dissected, immediately frozen in liquid nitrogen, and shipped to Shanghai Majorbio Bio-pharm Technology Co., Ltd. for intestinal fungal ITS region sequencing and microbiome analysis.

### 2.5 Growth indices and enzyme activity assays

The body weight of *A. custos* was measured using an electronic balance. First, each of the 15 *A. custos* in each of the five replicate groups was weighed individually. Subsequently, the average weight per *A. custos* within each replicate group was calculated. In the enzyme activity determination experiment, one intact insect was used as a single test sample for the determination of each indicator at each time point. Each treatment was subjected to three technical replicate measurements. Superoxide dismutase (SOD) activity was determined using an SOD assay kit based on the WST-8 method with spectrophotometry. Specifically, superoxide anions (O_2_^–^⋅) were generated via the xanthine-xanthine oxidase reaction system; these anions react with WST-8 to form a water-soluble formazan dye, which exhibits absorbance at 450 nm. SOD scavenges O_2_^–^⋅, thereby inhibiting formazan formation—with lighter yellow coloration of the reaction solution indicating higher SOD activity, and darker coloration indicating lower activity (Li et al., 2024). Catalase (CAT) activity was measured using a CAT assay kit with an ultraviolet spectrophotometer. Since H_2_O_2_ has a characteristic absorption peak at 240 nm, and CAT catalyzes H_2_O_2_ decomposition, the absorbance of the reaction solution at 240 nm decreases over time. CAT activity was calculated based on the rate of this absorbance decline (Deng et al., 2025). Peroxidase (POD) activity was assayed using a microbial POD kit with spectrophotometry. In the presence of H_2_O_2_, POD oxidizes guaiacol to form a tea-brown product, which shows maximum absorbance at 470 nm (Zheng et al., 2024). Glutathione S-transferase (GST) activity was determined using a GST assay kit with spectrophotometry. GST catalyzes the conjugation of glutathione (GSH) with 1-chloro-2,4-dinitrobenzene (CDNB), and the conjugated product exhibits an absorption peak at 340 nm. GST activity was calculated by measuring the rate of absorbance increase at 340 nm (Goggin et al., 2024). Acetylcholinesterase (AChE) activity was measured using an AChE kit with spectrophotometry. AChE catalyzes the hydrolysis of acetylcholine (Ach) to produce choline, which reacts with 5,5’-dithio-bis-(2-nitrobenzoic acid) (DTNB) to form 5-thio-2-nitrobenzoic acid (TNB). TNB has an absorption peak at 412 nm, and AChE activity was determined by the rate of absorbance increase at this wavelength (Lulić et al., 2025). Carboxylesterase (CarE) activity was assayed via visible light spectrophotometry following the manufacturer’s instructions for the CarE kit. CarE catalyzes the conversion of 1-naphthyl acetate to naphthol, which forms a colored complex with fast blue; CarE activity was calculated based on the rate of absorbance increase at 450 nm (Melchert et al., 2023). All enzyme assay kits used in this study were purchased from Enzyme-Linked Biotechnology Co., Ltd., (mlbio, Shanghai, China).

### 2.6 Extraction of ribosomal DNA (rDNA), PCR amplification, and sequencing of gut fungi in *Arma custos*

Total genomic DNA from the gut fungal community of *A. custos* was isolated using the E.Z.N.A.^®^ DNA Kit (Omega Bio-tek, Norcross, GA, United States.) following the manufacturer’s protocols. DNA integrity was assessed via 1% agarose gel electrophoresis, while its purity and concentration were quantified using a NanoDrop 2000 spectrophotometer. To amplify the variable ITS1 region of fungal ribosomal DNA (rDNA), PCR was performed with the primer pair ITS1F/ITS2R (Melchert et al., 2023; Chen et al., 2021). The 20 μL PCR system contained 4 μL of 5 × FastPfu Buffer, 2 μL of 2.5 mM dNTPs, 0.8 μL of each primer (5 μM), 0.4 μL of TransStart FastPfu DNA Polymerase (TransGen AP221–02), 0.2 μL of BSA, and 10 ng of template DNA. Amplification was conducted on an ABI GeneAmp 9700 thermal cycler (ABI, Carlsbad, CA) using the following program: initial denaturation at 95 °C for 3 min; 33 cycles of denaturation at 95 °C for 30 s, annealing at 55 °C for 30 s, and extension at 72 °C for 45 s; and a final extension at 72 °C for 10 min (Ma et al., 2023). PCR amplicons from each sample were pooled and separated by 2% agarose gel electrophoresis for target band recovery. The excised bands were purified using the AxyPrep DNA Gel Extraction Kit (Axygen Biosciences, Union City, CA, United States), with purification efficiency validated by 2% agarose gel electrophoresis. Purified DNA products were quantified using a Quantus™ Fluorometer (Promega, United States) (Ma et al., 2023). The gel image for PCR amplification result identification is shown in Supplementary Figure 1. After gel excision and purification of PCR products, libraries were constructed using the NEXTFLEX Rapid DNA-Seq Kit, sequencing was performed on an Illumina MiSeq platform to generate sequencing datasets. Raw ITS rDNA sequence data have been deposited in the NCBI Sequence Read Archive under BioProject ID: PRJNA1321102.

### 2.7 Bioinformatics statistics and analysis

The sequencing data were analyzed using QIIME2 (v2024^1^) on Majorbio’s I-Sanger BioCloud Platform. First, Fastp software (v0.23.4^2^) was employed to process the raw sequence data, after which Flash software (v1.2.11^3^) was used to splice the optimized data. The dada2 denoising and analysis pipeline (v2024, see text footnote 1) was then applied to remove duplicates and generate a table of amplicon sequencing variants (ASVs). For taxonomic analysis, the RDP classifier (v11.5) with a Bayesian algorithm was utilized; sequences were compared against the UNITE database at a confidence threshold of 0.7, with normalization based on the minimum number of sample sequences. Sequencing depth was standardized through rarefaction to the smallest sample size. Fungal functional annotations were determined by comparison using FUNGuild software^4^. Rectangular plots and line graphs were generated using OriginPro 2024, while Venn diagrams and neutral community model plots were created with R statistical tools. Beta diversity distance matrices were calculated via principal component analysis (PCA). Kaplan–Meier survival curve was generated using GraphPad Prism. To determine the significance of differences in survival rate, insect weight, and enzyme activity among different treatment groups, the Shapiro-Wilk test was first used to assess whether the data of each group conformed to a normal distribution. For datasets that met the normal distribution, one-way analysis of variance (ANOVA) was employed, followed by Duncan’s multiple range test for multiple comparisons; for data that did not conform to the normal distribution, the Kruskal-Wallis *H*-test was used for analysis. Statistical analyses were performed using IBM SPSS Statistics 23 for Windows.

## 3 Results

### 3.1 Differences in growth status and enzyme activity of *Arma custos* under different pesticide treatments

#### 3.1.1 Body weight changes and survival rates of *Arma custos* in different treatment groups

**FIGURE 1:**
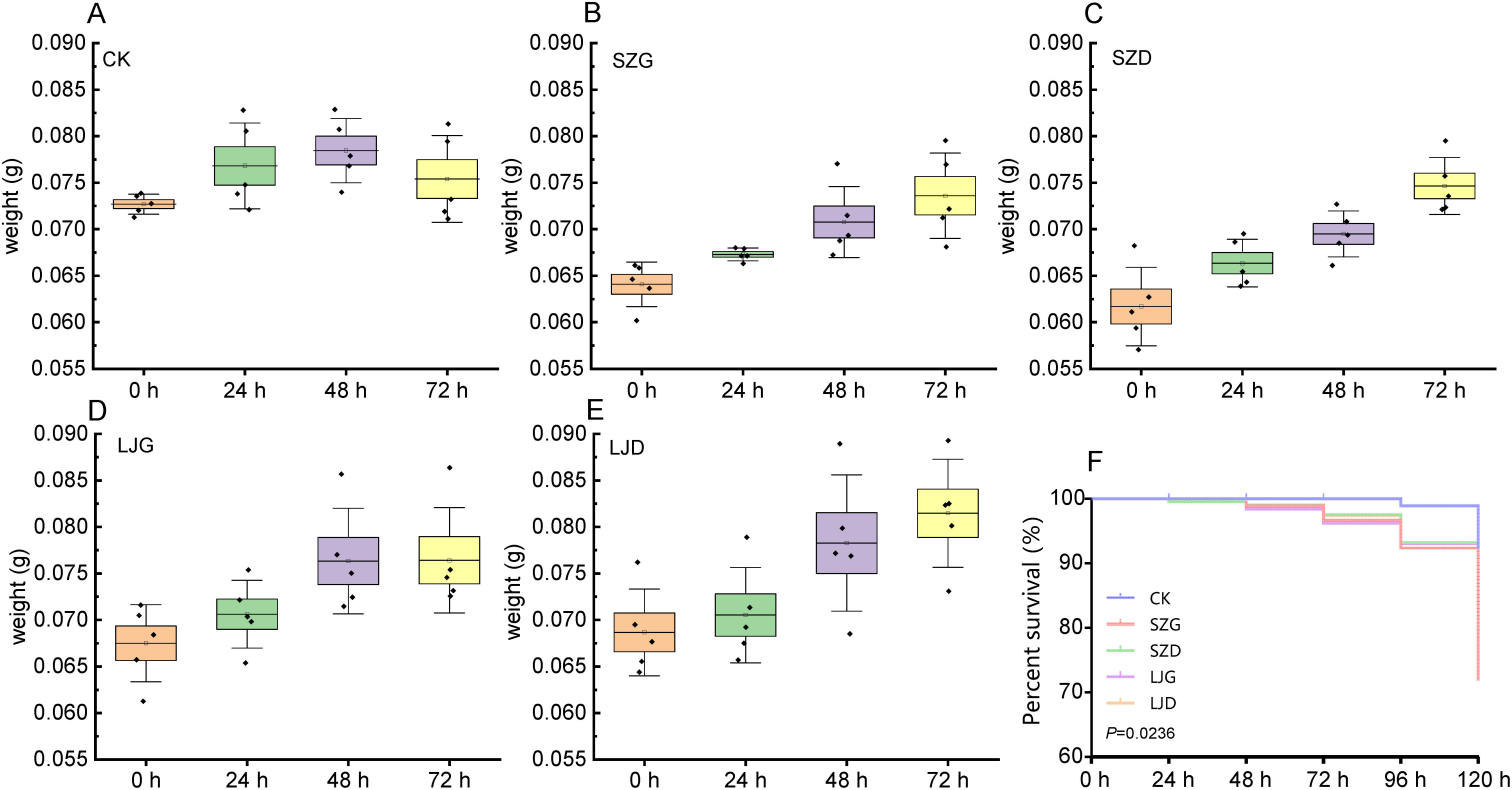
Changes in body weight and survival probability curves of *A. custos* under different pesticide treatments. **(A–E)** Show temporal changes in the average body weight of each technical replicate of *A. custos* at 0, 24, 48, and 72 h post-treatment under the control (CK) and pesticide treatments, respectively (*n* = 5). One-way ANOVA results for inter-temporal differences in body weight are as follows: CK group (*F* = 2.122, df = 3, 16; *p* > 0.05), SZG (*F* = 8.177, df = 3, 16; *p* < 0.05), SZD (*F* = 14.894, df = 3, 16; *p* < 0.05), LJG (*F* = 4.105, df = 3, 16; *p* < 0.05), and LJD (*F* = 5.545, df = 3, 16; *p* < 0.05). **(F)** Presents the survival rate curves of *A. custos* under these treatments within 120 h.

To elucidate the effects of different treatments on *A. custos* weight dynamics and survival, we conducted comprehensive analyses using boxplots and a Kaplan–Meier survival curve (Figure 1). In the control group (CK, Figure 1A), no significant variation in body weight was observed within 72 h (*p* > 0.05), with the average weight increasing from 0.0727 to 0.0754 g, corresponding to a growth rate of 3.72%. In the SZG group (Figure 1B), body weight changes within 72 h were significant (*p* < 0.05), with the average weight rising from 0.0641 to 0.0736 g (14.84% growth rate). Similarly, the SZD group (Figure 1C) exhibited significant weight gains over 72 h (*p* < 0.05), with the average weight increasing from 0.0617 to 0.0747 g, representing a 21.07% growth rate. For the LJG group (Figure 1D), body weight changes within 72 h were non-significant (*p* > 0.05), with the average weight increasing from 0.0675 to 0.0764 g (13.18% growth rate). In the LJD group (Figure 1E), significant weight changes were detected within 72 h (*p* < 0.05), with the average weight growing from 0.0687 to 0.0815 g (18.63% growth rate). At 72 h post-treatment, the body weight growth rates of all treatment groups were higher than that of the control group (CK group): among them, the body weight growth rates of the SZG group and SZD group reached 3.99 times and 5.65 times that of the CK group, respectively, while those of the LJG group and LJD group reached 3.55 times and 5.02 times that of the CK group, respectively.

The Kaplan - Meier survival curve (Figure 1F) illustrates the proportion of survivors in different groups over time, and there were significant differences in survival rates among the groups (*p* < 0.05). The survival curve of the CK group was consistently at the highest level, and the survival rate was still close to 100% at 120 h, indicating that the survival status of the experimental subjects in the control group was stable. The survival curve of the SZG group declined the earliest and most drastically, and the survival rate in the later stage was significantly lower than that of other groups, suggesting that the SZG treatment had the most prominent negative impact on the survival of the experimental subjects. The survival curves of the SZD and LJG groups declined more gently than that of the SZG group, and the gap with the CK group was smaller than that of the SZG group, indicating that these two treatments had relatively mild inhibitory effects on survival. The survival curve of the LJD group was most similar to that of the CK group, and the survival rate in the later stage was only slightly lower than that of the CK group. Combined with the body weight data, it suggested that the LJD treatment might be more conducive to maintaining the survival status of the experimental subjects in the “survival - growth” trade - off.

#### 3.1.2 Differences in enzyme activity among different treatment groups

**FIGURE 2:**
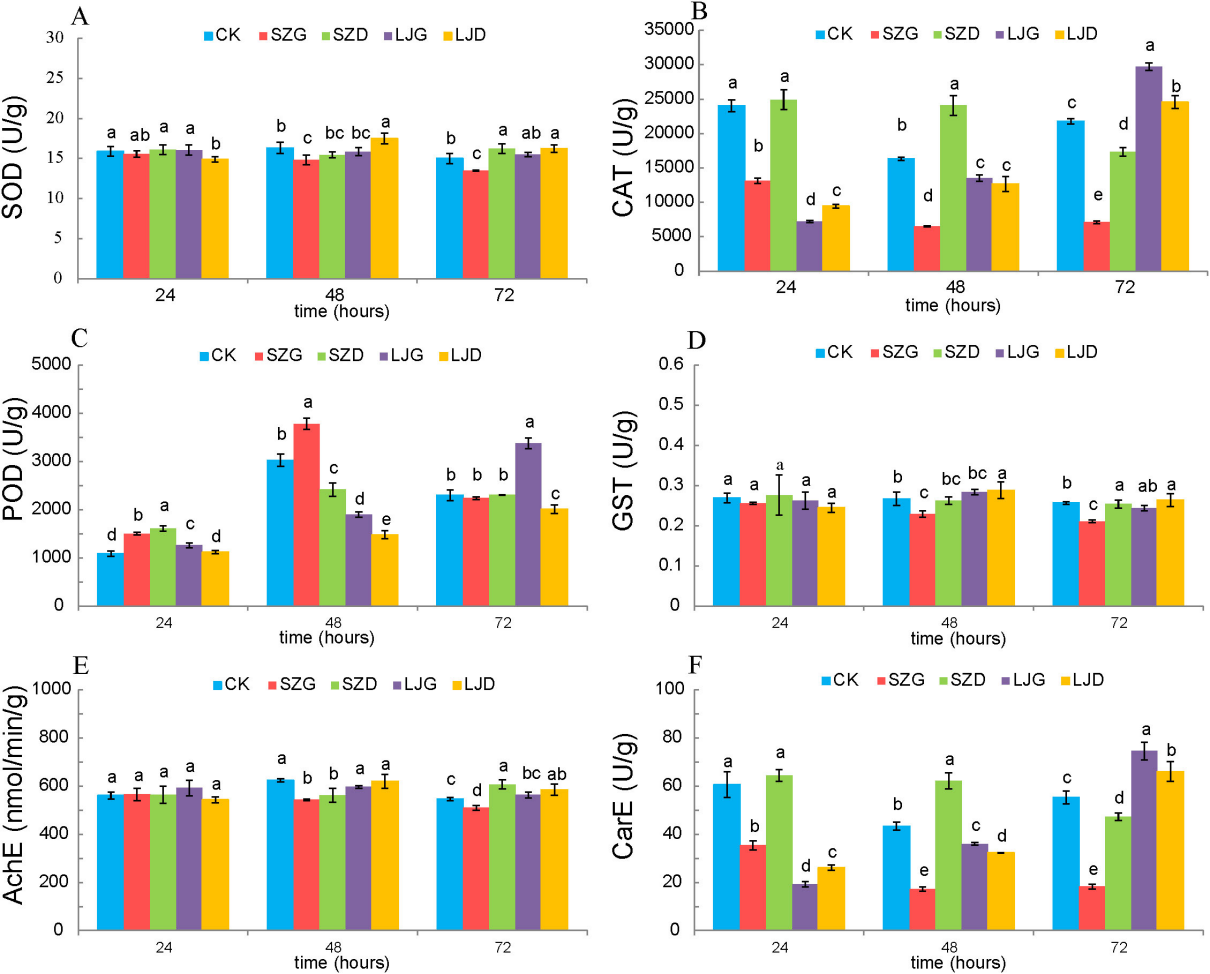
Comparison of enzyme activities in *A. custos* under different pesticide treatments. In the figure, **(A)** SOD: 24 h (*F* = 2.850, df=̃ 4, 10; *p* > 0.05), 48 h (*F* = 8.716, df=̃ 4, 10; *p* < 0.05), 72 h (*F* = 17.981, df=̃ 4, 10; *p* < 0.05); **(B)** CAT: 24 h (*H* = 12.833, df=̃ 4; *p* < 0.05), 48 h (*F* = 168.309, df=̃ 4, 10; *p* < 0.05), 72 h (*F* = 629.474, df=̃ 4, 10; *p* < 0.05); **(C)** POD: 24 h (*F* = 75.089, df=̃ 4, 10; *p* < 0.05), 48 h (*F* = 205.069, df=̃ 4, 10; *p* < 0.05), 72 h (*H* = 11.7, df=̃ 4; *p* < 0.05); **(D)** GST: 24 h (*H* = 4.282, df=̃ 4; *p* > 0.05), 48 h (*F* = 9.201, df=̃ 4, 10; *p* < 0.05), 72 h (*F* = 15.563, df=̃ 4, 10; *p* < 0.05); **(E)** AChE: 24 h (*F* = 1.403, df=̃ 4, 10; *p* > 0.05), 48 h (*F* = 10.442, df=̃ 4, 10; *p* < 0.05), 72 h (*F* = 17.188, df=̃ 4, 10; *p* < 0.05); **(F)** CarE: 24 h (*F* = 157.269, df=̃ 4, 10; *p* < 0.05), 48 h (*F* = 266.440, df=̃ 4, 10; *p* < 0.05), 72 h (*F* = 172.840, df=̃ 4, 10; *p* < 0.05). Different lowercase letters denote significant differences among different pesticide treatments at *P* < 0.05. The maximum values in each column are denoted by “a” (*n* = 3).

To investigate the effects of different treatments on enzyme activities in *A. custos*, we analyzed the activities of SOD, CAT, POD, GST, AChE, and CarE across three time points (24, 48, and 72 h) in the control group (CK) and treatment groups (SZG, SZD, LJG, LJD). One-way analysis of variance was applied to data that satisfied the normality distribution assumption, while the Kruskal-Wallis *H*-test was used for data deviating from normality, to determine the significance of differences in enzyme activities among the various treatment groups (Figure 2). For SOD activity (Figure 2A), the control group (CK) exhibited significantly higher activity than the SZG at 48 h (*p* < 0.05), with this significant difference persisting at 72 h (*p* < 0.05). Regarding CAT activity (Figure 2B), CK showed significantly higher activity compared to SZG at 24 h (*p* < 0.05), and this trend remained consistent at 48 and 72 h (*p* < 0.05). In contrast, for POD activity (Figure 2C), CK displayed significantly lower activity than SZG at 24 h (*p* < 0.05), with the same pattern observed at 48 and 72 h (*p* < 0.05). For GST activity (Figure 2D), CK had significantly higher activity than SZG at 48 h (*p* < 0.05), and this significant difference was maintained at 72 h (*p* < 0.05). Similarly, AChE activity (Figure 2E) in CK was significantly higher than that in SZG at 48 h (*p* < 0.05), with the difference remaining significant at 72 h (*p* < 0.05). For CarE activity (Figure 2F), CK exhibited significantly higher activity compared to SZG at 24 h (*p* < 0.05), and this significant disparity persisted at 48h and 72 h (*p* < 0.05). Collectively, these results indicate that SZG treatment markedly altered the activities of most enzymes relative to CK. In contrast, the SZD, LJG, and LJD groups had relatively minor impacts, as their enzyme activities did not show consistent, significant differences compared to CK across all tested time points and enzyme types.

### 3.2 RNA sequencing and community composition analysis of gut fungi in *Arma custos*

#### 3.2.1 Comparison of RNA sequencing results among different treatment groups

**FIGURE 3:**
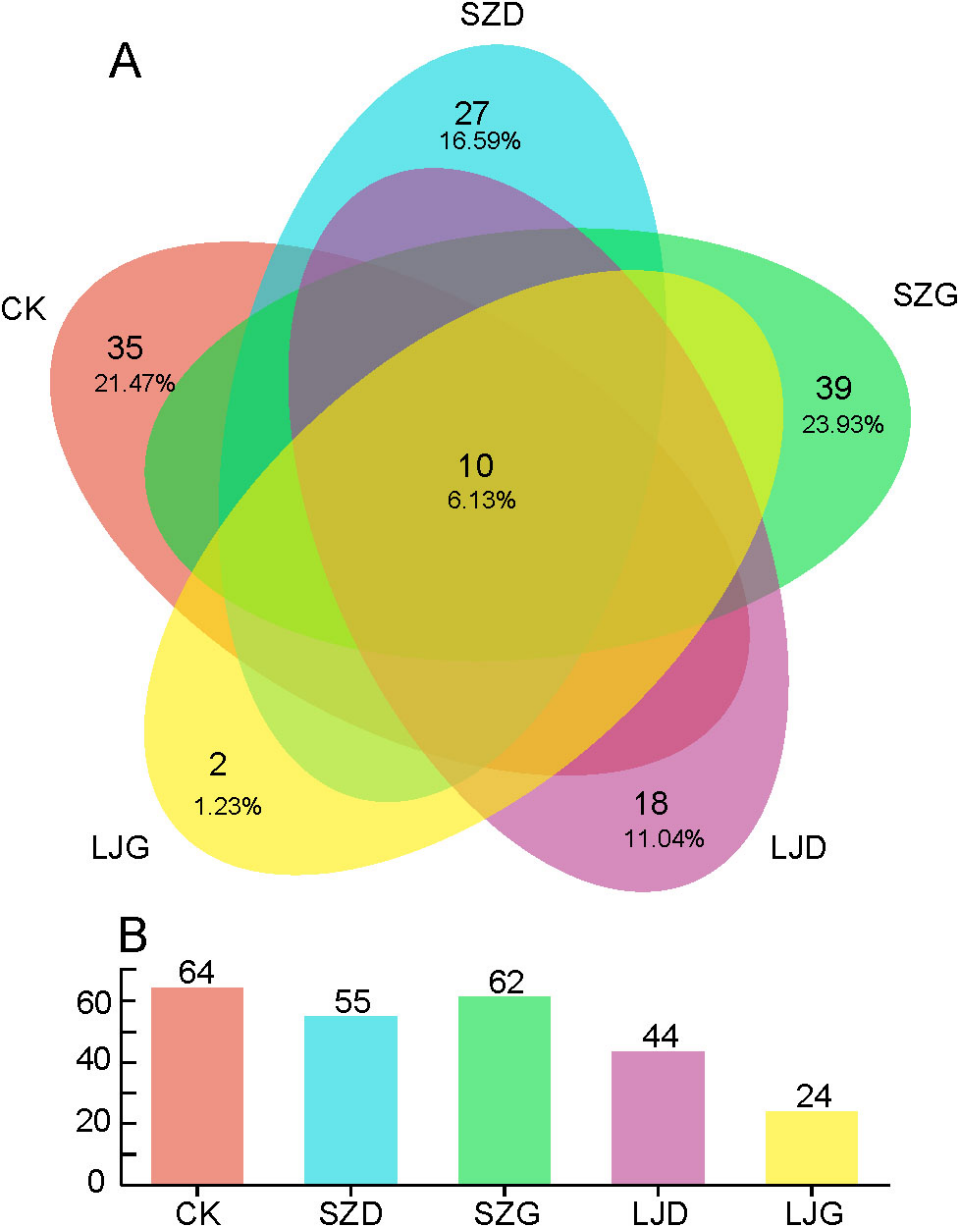
Comparison of the number of intestinal fungal amplicon sequencing variants (ASVs) in *A. custos* under different pesticide treatments. This image consists of a Venn diagram **(A)** and a bar chart **(B)**, illustrating the distribution and quantity of elements related to different categories (CK, SZD, SZG, LJD, LJG). In the Venn diagram, each colored ellipse represents a different category, and the overlapping areas show the common elements among categories along with their proportions (*n* = 3).

Gut fungi of *A. custos* were analyzed via MiSeq high-throughput sequencing (Supplementary Table 1). A total of 753,214 raw sequences were generated from 15 gut samples across five treatment groups, yielding 225,964,200 total base pairs (bp) with a paired-end (PE) 300 bp sequencing strategy. Following quality filtering, 739,795 high-quality sequences remained, comprising 162,664,825 bp with an average read length of 226 bp. As illustrated in Figure 3A, a total of 159 fungal amplicon sequence variants (ASVs) were identified across all 15 treated gut samples (Supplementary Table 2). Among these, ten ASVs were shared across all treatment groups, accounting for ∼6.13% of the total. The SZG group exhibited the highest number of unique ASVs (39), representing ∼23.93% of all detected ASVs, while the LJG group harbored the fewest unique ASVs (2, ∼1.23%). The CK, SZD, and LJD groups comprised 21.47%, 16.59%, and 11.04% of unique ASVs, respectively. Overall, the number of ASVs in the SZ group was similar to that in the CK group, while the number of ASVs in the LJ group was significantly lower than that in the CK group (Figure 3B).

#### 3.2.2 Differences in fungal genus composition and indicator species

**FIGURE 4:**
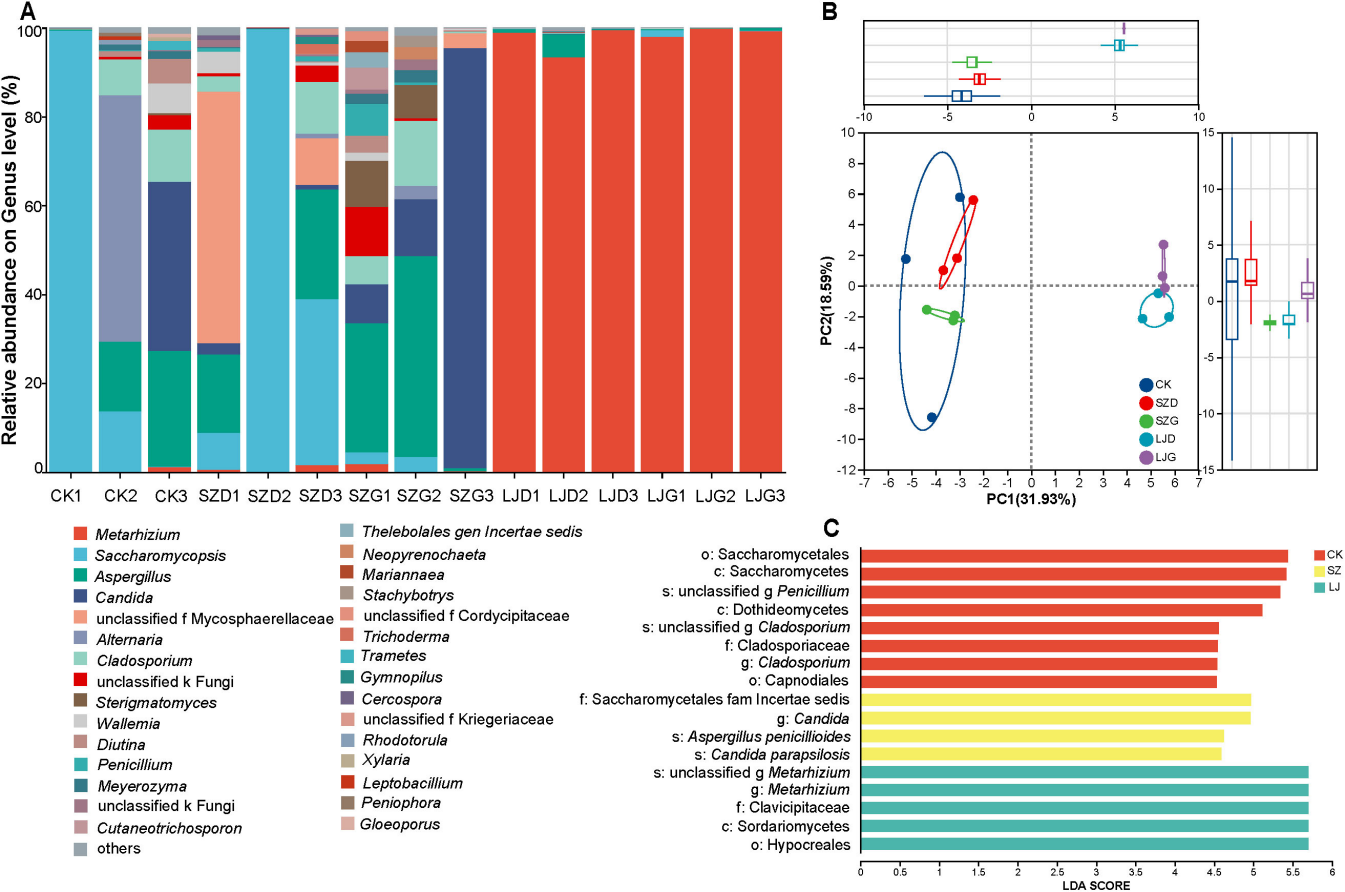
Differences in intestinal fungal community structure and indicator species of *A. custos* under different pesticide treatments. This image is composed of three parts **(A–C)** to analyze the fungal community structure. **(A)** A stacked bar chart displays the relative abundance of different fungal genera across various samples. Each color represents a specific genus, enabling the visualization of genus - level composition differences among samples. **(B)** A principal component analysis (PCA) plot, constructed using Bray - Curtis distances, demonstrates the segregation of fungal communities across different groups. **(C)** A linear discriminant analysis (LDA) effect size (LEfSe) plot (with LDA score threshold > 4) identifies microbial taxa with significant differences in abundance among groups, highlighting key genera contributing to community distinctions (*n* = 3).

Taxonomic analysis of the representative ASV sequences of fungi revealed that a total of 4 phyla, 14 classes, 29 orders, 50 families, and 50 genera of fungi were detected in the 15 samples. As shown in Figure 4, at the genus level (Figure 4A), *Metarhizium dominated the gut in the LJ group, along with Saccharomycopsis and Aspergillus, suggesting that M. anisopliae had fully invaded the gut*. In the CK and SZ groups, the dominant gut fungi were *Saccharomycopsis*, *Aspergillus*, and *Candida*; in some individual samples, *Alternaria*, *Sterigmatomyces*, *Cladosporium*, and *Wallemia* also exhibited significantly dominant relative abundances. Principal component analysis (PCA) based on the Pearson distance algorithm was used to compare the similarities and differences in fungal communities among different treatments (Figure 4B). The gut fungal communities of the CK and SZ groups were located on the negative half-axis of the PC1, while those of the LJ group were on the positive half-axis of the PC1. The gut fungal communities of the CK and SZ groups were positioned on the negative half - axis of PC1, while those of the LJ group were on the positive half - axis of PC1. The ANOSIM inter - group difference test revealed that the gut fungal community structures of the CK and SZ groups were similar but significantly different from that of the LJ group (*p <* 0.05).

A non-parametric Kruskal-Wallis rank sum test was employed to assess significant differences in fungal taxa composition across distinct taxonomic levels among the treatment groups (Figure 4C). A total of 17 taxa satisfied the predefined screening criterion (*LDA* score > 4.0), with detailed effect sizes and corresponding p-values provided in Table 1.

**TABLE 1:** Linear discriminant analysis (LDA) discriminant results.

Group	Species name	Mean	LDA value	*P*-value
CK	o: Saccharomycetales	5.730253	5.438824	0.006251
CK	g: *Penicillium*	3.024849	5.34373	0.013764
CK	o: Capnodiales	4.822527	4.530448	0.029175
CK	c: Dothideomycetes	5.401189	5.116987	0.040067
CK	c: Saccharomycetes	5.730253	5.419901	0.006251
CK	g: *Cladosporium*	4.822527	4.540763	0.029175
CK	f: Cladosporiaceae	4.822527	4.546645	0.029175
CK	g: *Cladosporium*	4.822527	4.558765	0.024827
SZ	s: *Candida parapsilosis*	4.339535	4.593504	0.006451
SZ	g: *Candida*	5.300288	4.964632	0.034884
SZ	s: *Aspergillus penicillioides*	4.755003	4.623718	0.044243
SZ	f: Saccharomycetales fam Incertae sedis	5.314574	4.967314	0.011695
LJ	g: *Metarhizium*	5.991859	5.703193	0.005148
LJ	o: Hypocreales	5.991898	5.697437	0.0058
LJ	c: Sordariomycetes	5.99192	5.699586	0.0058
LJ	f: Clavicipitaceae	5.991859	5.70283	0.005148
LJ	g: *Metarhizium*	5.991859	5.703018	0.005148

At the class level, Saccharomycetes and Dothideomycetes were identified as significantly dominant taxa in the CK group, whereas Sordariomycetes exhibited dominance in the LJ group (*p* < 0.05). At the order level, Saccharomycetales and Capnodiales were the dominant taxa in the CK group, while Hypocreales dominated the LJ group (*p* < 0.05). For the family level, Cladosporiaceae (CK group), Clavicipitaceae (LJ group), and Saccharomycetales fam. Incertae sedis (SZ group) were recognized as significantly dominant (*p* < 0.05). At the genus level, the dominant taxa included *Cladosporium* (CK group), *Candida* (SZ group), and *Metarhizium* (LJ group) (*p* < 0.05). Finally, at the species level, *Cladosporium halotolerans* (CK group), *Aspergillus aflatoxiformans* (LJ group), as well as *Candida parapsilosis* and *Aspergillus penicillioides* (SZ group) were confirmed as significantly dominant taxa (*p* < 0.05).

### 3.3 Differences in gut fungal functions of *Arma custos* under different pesticide treatments

**FIGURE 5:**
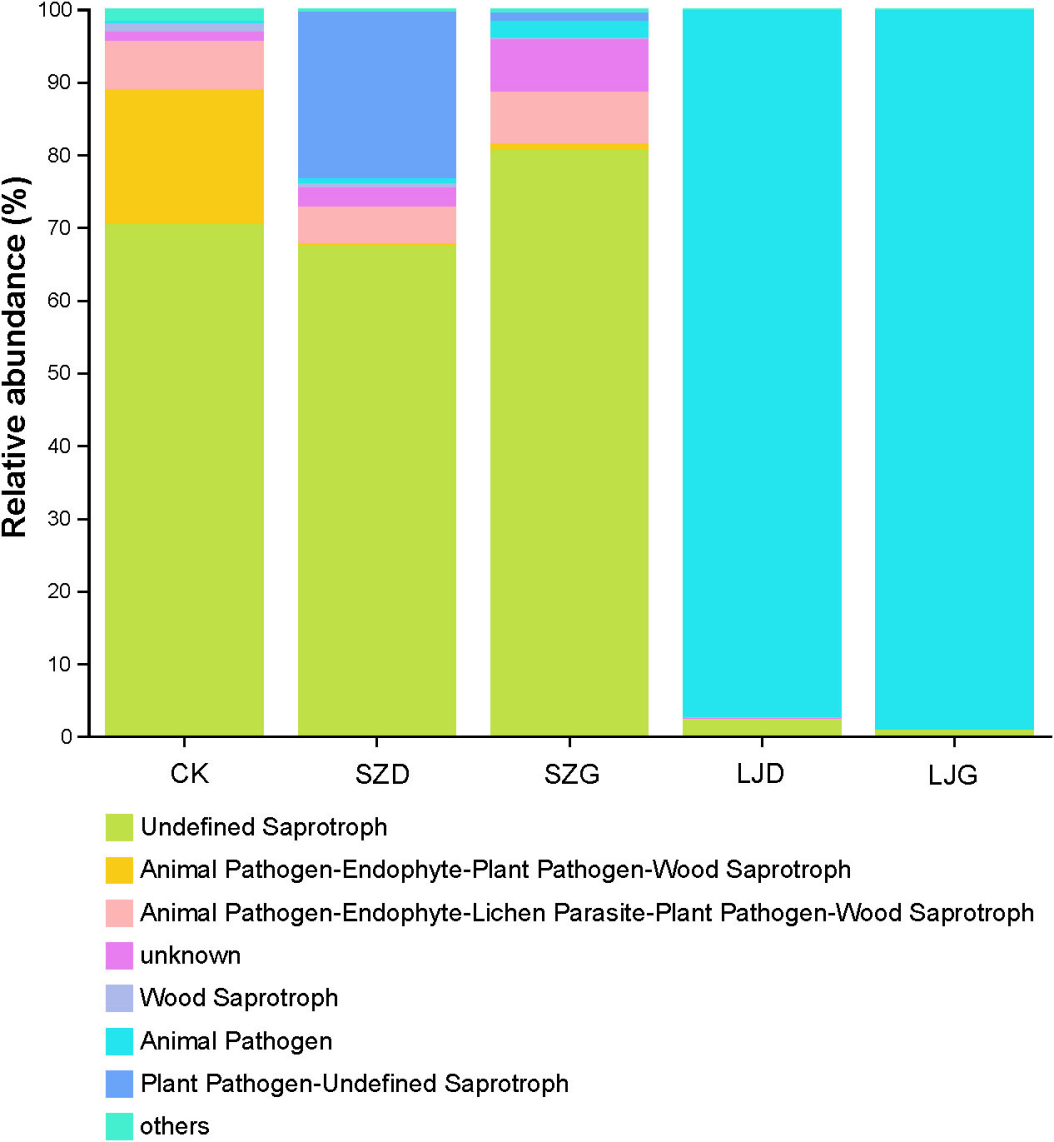
Functional changes of intestinal fungi in *Arma custos* under different pesticide treatments. This stacked bar chart illustrates the relative abundance (%) of different fungal functional groups across five categories: CK, SZD, SZG, LJD, and LJG. Each color represents a specific functional group (*n* = 3).

Functional profiling of fungal communities with respect to resource utilization pathways was conducted using the FUNGuild microecology tool (Nguyen et al., 2016). As illustrated in Figure 5, detected fungi were classified into three trophic modes based on their environmental resource utilization strategies: symbiotrophs, pathotrophs, and saprotrophs, encompassing 12 functional categories. These included litter saprotrophs, soil saprotrophs, wood saprotrophs, bryophyte parasites, lichen parasites, ericoid mycorrhizal fungi, ectomycorrhizal fungi, animal pathogens, dung saprotrophs, plant pathogens, endophytes, and fungal parasites.

In the CK group, the Undefined Saprotroph (confidence level: Possible) was predominant, accounting for over 70% of the relative abundance. Additionally, other functional guilds, such as “Animal Pathogen - Endophyte - Plant Pathogen - Wood Saprotroph” and “Animal Pathogen - Endophyte - Lichen Parasite - Plant Pathogen - Wood Saprotroph,” were also detected at relatively low proportions. For the SZD treatment, a distinct shift in the fungal functional community was observed. The “Plant Pathogen - Undefined Saprotroph” (confidence level: Possible) guild showed a notable increase compared to the CK group, while the relative abundance of “Undefined Saprotroph” (confidence level: Possible) decreased. In the SZG group, the “Undefined Saprotroph” (confidence level: Possible) guild still maintained a high proportion (exceeding 80%), but the contributions from other functional guilds were higher than those in the CK group. Notably, the LJD and LJG groups exhibited a dramatically different functional composition. The “Animal Pathogen” (confidence level: Possible) guild overwhelmingly dominated, with a relative abundance approaching 100%, completely altering the saprotrophic-dominated structure seen in the CK group. These results, based on FUNGuild predictions, suggest that different treatments may impose distinct selective pressures on the fungal community, potentially leading to variations in the relative abundance of predicted fungal trophic modes. However, it should be noted that FUNGuild assignments are inherently predictive and coarse. Detailed results can be referred to in Supplementary Table 3.

### 3.4 Relationship between growth status, enzyme activity, and gut fungal community of *Arma custos*

**FIGURE 6:**
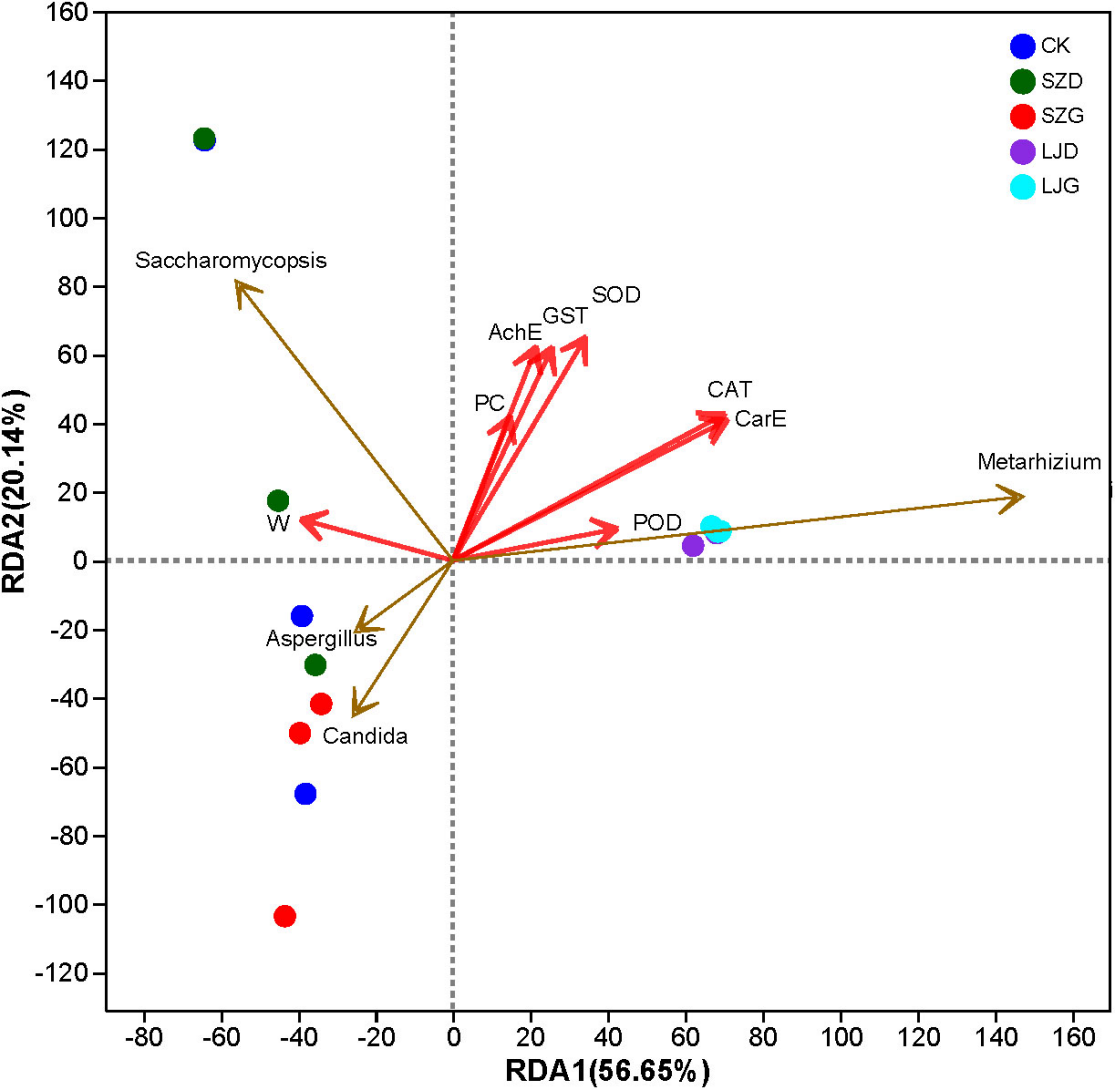
Redundancy analysis (RDA) of growth status, enzyme activity, and gut fungal community of *A. custos.* This is a redundancy analysis (RDA) plot showing the relationships between fungal genera (*Saccharomycopsis*, *Aspergillus*, *Metarhizium*) and various variables (AchE, GST, SOD, PC, CAT, CarE, W, POD) across different groups (CK, SZD, SZG, LJD, LJG). Red arrows represent the physiological index variables, with their directions and lengths indicating the strength and direction of correlations, and blue arrows represent the microbial communities that play a key role. Points represent samples from different groups, and the positions reflect the associations between fungal genera, physiological index variables, and group differences.

Redundancy analysis (RDA) was performed to explore relationships between the gut fungal community and growth-physiological indices of *A. custos*, with results presented in Figure 6. The first and second axes collectively explained 76.79% of the total variance, confirming their capacity to capture associations between growth-physiological status and gut fungal community structure. Permutation tests indicated that the gut fungal community structure significantly drove variations in CAT (*r*^2^ = 0.6812, *p* = 0.002), CarE (*r*^2^ = 0.6775, *p* = 0.003), SOD (*r*^2^ = 0.5105, *p* = 0.024), GST (*r*^2^ = 0.4182, *p* = 0.041), and AchE (*r*^2^ = 0.3998, *p* = 0.047) activities. The abundance of *M. anisopliae* was positively correlated with the activities of catalase (CAT), carboxylesterase (CarE), superoxide dismutase (SOD), glutathione S-transferase (GST), and acetylcholinesterase (AChE) in *A. custos* (*p <* 0.05). Conversely, tetraniliprole was negatively correlated with CAT and CarE activities (*p <* 0.05). *Saccharomycopsis* displayed significant dominance in select samples, which corresponded to alterations in growth-physiological indices. Notably, *Metarhizium* had no significant impact on POD (*r*^2^ = 0.1843, *p* = 0.251), PC (*r*^2^ = 0.1765, *p* = 0.326), or W (*r*^2^ = 0.1596, *p* = 0.359) in *A. custos*.

### 3.5 Mechanism underlying the gut fungal community assembly in *Arma custos*

**FIGURE 7:**
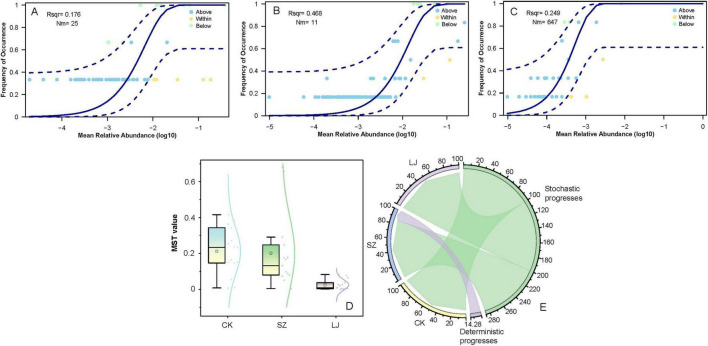
Gut fungal community assembly of *A. custos* based on neutral community model and modified stochasticity ratio. This image consists of five subfigures **(A–E)** analyzing microbial community dynamics. **(A–C)** These are occupancy - abundance relationship plots. They show the frequency of occurrence of microbial taxa against their mean relative abundance (log10 - transformed). Different lines and points represent taxa “Above,” “Within,” and “Below” certain abundance thresholds, with parameters like Rsq and Nm provided for model fitting. **(D)** A box plot displays the MST (Microbial Stability Trait) values across three groups (CK, SZ, LJ), illustrating differences in microbial stability among them. **(E)** A circular plot depicts the contributions of deterministic and stochastic processes to microbial community assembly for groups CK, SZ, LJ, and others, with the arc length indicating the extent of each process.

To explore the distribution patterns and assembly mechanisms of microbial communities under different treatments, we first analyzed the relationship between mean relative abundance and frequency of occurrence for microbial taxa (Figure 7A–C). In each panel, the solid curves represent the fitted relationships, while the dashed lines denote the 95% confidence intervals. For the CK group (Figure 7A, *r*^2^ = 0.176), taxa with relatively low mean relative abundance showed a gradual increase in occurrence frequency, while those with higher abundances tended to stabilize at a high occurrence rate. For the SZ group (Figure 7B, *r*^2^ = 0.468), a stronger positive correlation between abundance and occurrence was observed, indicating a more pronounced tendency for abundant taxa to be widely distributed. The LJ group (Figure 7C, *r*^2^ = 0.249) exhibited an intermediate pattern, with a positive but less robust association compared to SZ.

We further quantified microbial stochasticity using the MST value, which reflects the extent of stochastic processes in community assembly. As shown in Figure 7D, the MST values differed significantly among CK (MST = 0.2128 ± 0.1306), SZ (MST = 0.2012 ± 0.2041), and LJ (MST = 0.0246 ± 0.0284) groups (*F* = 21.237, df = 2, *p* < 0.001). The CK group had the highest median MST value, followed by SZ, while LJ exhibited the lowest MST value. This pattern suggested that stochastic processes dominated community assembly in CK, with deterministic processes gaining more influence in SZ and becoming the most prominent driver in LJ. Such shifts indicated that environmental filtering or biotic interactions (deterministic factors) played an increasingly important role in shaping fungal communities from CK to LJ.

To visualize the relative contributions of deterministic and stochastic processes, we constructed a circular plot (Figure 7E). The CK group showed the largest sector for “Stochastic progresses” (accounting for a substantial proportion), confirming that stochastic events (e.g., dispersal limitation, ecological drift) were the primary drivers of community assembly. In contrast, the LJ group had a much smaller stochastic sector and a notable “Deterministic progresses” component (with a specific proportion marked). The SZ group exhibited an intermediate pattern, with contributions from both processes but leaning more toward stochasticity than LJ. These results collectively demonstrated that the mechanism of fungal community assembly shifted from stochastic - dominated in CK to deterministic - dominated in LJ, with SZ representing a transitional state.

## 4 Discussion

Growth trajectories and physio-biochemical traits respond to environmental stressors is not only pivotal for unraveling the stability of natural enemies’ pest-control functions but also provides a critical basis for assessing pesticide toxicity and safety (Liu et al., 2023; Mustafa et al., 2024). Thus, this study represents the first investigation into the effects of two novel pesticides—tetraniliprole and *Metarhizium*—on the physiological indices and growth performance of the predatory bug *Arma custos*. By further exploring their regulatory impacts on the structure and function of *A. custos* gut fungal communities, this research aims to comprehensively evaluate the potential implications of these pesticides for predatory insects.

In recent years, an accumulating body of research has revealed that pesticide contaminants differentially impact growth parameters of predatory insects across developmental stages (Camerini et al., 2024; Calvo-Agudo et al., 2019). Notably, hormetic effects—where low-dose agrochemical exposure enhances biological functions—have been documented to boost reproductive capacity, extend lifespan, elevate survival rates, and enhance insect immunity (Li et al., 2023). For example, quizalofop-p-ethyl (QpE) induces hormesis in *Propylea japonica*, significantly increasing survival rates of F2 and F3 females, F3 males, and body weight in F3 males under continuous exposure (Chang et al., 2023). These phenomena reflect adaptive mechanisms in insects, whereby environmental perturbations drive increased food intake and energy storage to mitigate stress (Levin, 2006). Our study aligns with this paradigm: *A. custos* exposed to SZ or LJ exhibited 72 h body weight growth rates exceeding 10%, compared to 3.72% in unexposed controls. This suggests both pesticides promote weight gain in predatory insects. However, neither SZ nor LJ improved survival rates. After 120 h of SZ exposure, *A. custos* survival dropped to 78.67%, while LJ exposure reduced survival to 85.33%—both significantly lower than the control’s > 90% survival. From a fitness perspective, SZ and LJ likely impose adverse effects on predatory insects. Reduced survival directly reflects stress sensitivity, whereas weight gain—beneficial under controlled rearing—may impair natural defenses and foraging efficiency (Tokach et al., 2024; Colin et al., 2019). A critical unresolved question remains: Can accelerated weight growth persist under natural food-limited conditions? Further research is needed to address this ecological relevance.

Enzyme activities in insects are pivotal for regulating growth, metabolism, and stress resilience (Liu et al., 2023). Prior research has demonstrated that escalating phoxim concentrations enhance glutathione S-transferase (GST) activity by up to 2.3-fold in *Pealius mori*, while inhibiting acetylcholinesterase (AchE) activity to 30% of control levels—confirming both the neurotoxic properties of organophosphates and the induction of GST as an adaptive detoxification mechanism (Sheng et al., 2021). Consistent with these findings, our study revealed that 72 h exposure to SZG significantly suppressed superoxide dismutase (SOD), GST, and AchE activities relative to other treatments (*P* < 0.05). This dual inhibition of neural targets and antioxidant enzymes is likely mediated by the electrophilic trifluoromethyltetrazole moiety in tetraniliprole. As observed with other diamide insecticides (e.g., flubendiamide), this structural feature may covalently interact with cysteine thiols in the GST active site or competitively displace glutathione (GSH) binding, thereby impairing detoxification function (Zhao et al., 2020). In contrast, LJ exposure significantly elevated SOD, GST, and AchE activities (*P* < 0.05) compared to controls, reflecting an active defensive response in *A. custos*. Augmented SOD activity likely mitigates oxidative damage by scavenging toxic hydroxyl radicals, while enhanced GST activity facilitates conjugation and excretion of LJ-derived metabolites, reducing their bioavailability (Baranova et al., 2024). Redundancy analysis further validated the correlation between the pesticide and enzyme activities, revealing a positive correlation between *Metarhizium* and the activities of catalase (CAT), carboxylesterase (CarE), superoxide dismutase (SOD), glutathione S-transferase (GST), and acetylcholinesterase (AChE)—all of which are key enzymes involved in the detoxification of xenobiotics.

Gut microbial communities play a pivotal role in maintaining health and mediating disease resistance in predatory insects, exhibiting adaptive plasticity in response to diverse feeding strategies and environmental fluctuations (Xie et al., 2024; Huang et al., 2021). Our results revealed that a total of 159 amplicon sequence variants (ASVs) were detected across 15 samples, a number that is significantly low for an ITS sequencing dataset of insect gut microbiota. This observation is biologically plausible when considering the growth and developmental characteristics of *A. custos*. Previous studies have confirmed that the gut microbial diversity of *A. custos* gradually increases throughout its growth and development, peaking at the adult stage (Li et al., 2022). This diversity accumulation process relies on the insect’s exposure to a broader external environment and ingestion of more diverse food resources. The *A. custos* samples used in this experiment were 5th-instar nymphs, a stage that has not yet encountered complex external environments. Their gut microbiota remains in a “species accumulation phase” with immature community structure, and thus the relatively low microbial richness detected at this stage is consistent with their biological developmental patterns. Our findings reveal that the gut fungal community assembly of *A. custos* is governed by deterministic processes driven by external factors, underscoring the key roles of pesticide toxicity and exogenous fungi in shaping gut fungal community structure. In the LJ group, *Metarhizium* dominated the gut microbiota, accompanied by *Saccharomycopsis* and *Aspergillus*—a pattern indicative of complete gut colonization by *M. anisopliae*. Concurrently, fungal species richness in the LJ group was lower than in SZ and CK groups, suggesting LJ may suppress the growth of other fungal taxa. This suppression likely facilitates the proliferation of drug-resistant pathogens, leading to microbial dysbiosis and reduced community diversity. In the CK and SZ groups, *Saccharomycopsis*, *Aspergillus*, and *Candida* emerged as dominant taxa, with *Alternaria*, *Sterigmatomyces*, *Cladosporium*, and *Wallemia* exhibiting significantly high relative abundances in select samples. Nonparametric Kruskal-Wallis rank-sum tests identified *Candida parapsilosis* and *Aspergillus penicillioides* as signature fungal taxa in the SZ group, reflecting their robust adaptation to SZ-induced stress. In contrast, *Cladosporium halotolerans* was the dominant signature taxon in the CK group—a ubiquitous fungal lineage primarily acquired from dietary and environmental sources (Kaur et al., 2023).

Recent investigations have underscored that insect-associated microbial symbionts play a critical role in protecting hosts against toxic insults (van den Bosch and Welte, 2017; Wang et al., 2025). However, their community structures and functions are frequently perturbed by environmental stressors such as pesticides, thereby threatening the health of predatory insects (Hotchkiss et al., 2024). Predictive functional profiling of gut fungi using the FUNGuild microecology tool revealed that *M. anisopliae* belongs to the animal pathogen guild (confidence category: Possible). The marked proliferation of these pathogenic fungi not only impairs the microbiota’s nutritional support and immunomodulatory functions but also induces host metabolic disorders and immune suppression. Consequences include reduced survival, fecundity, and predatory capacity of predatory insects, thereby compromising their pest-control efficacy in agroecosystems (Jirešová et al., 2025). In the CK group, gut fungi were predominantly saprotrophic, engaging in metabolic synergism with the host to facilitate digestion of complex nutrients, vitamin biosynthesis, and energy absorption—processes vital for normal growth and development (Lambert et al., 2024). Conversely, the SZ group exhibited elevated proportions of plant and animal pathogens (confidence category: Possible), indicative of a complex feedback loop between pathogenic flora imbalance and host health risks (Hsu and Schnabl, 2023). Microbiota dysregulation may exacerbate host deterioration via mechanisms such as intestinal microenvironment alteration and inflammatory responses, while declining host health further destabilizes the microbial community, forming a vicious cycle (Farini et al., 2023). Future research should aim to elucidate the tripartite interaction mechanisms among microorganisms, hosts, and environmental stressors, while investigating microbial modulation strategies to restore pesticide-disrupted gut microbiota. Such efforts will provide both theoretical foundations and practical solutions for safeguarding predatory insect health and enhancing biological control sustainability (El Tekle and Garrett, 2023).

## 5 Conclusion

Different treatments impacted the microbial community structure, phylogenetic turnover, and assembly processes. The LJ treatment reduced phylogenetic turnover and enhanced the role of deterministic processes in community assembly, whereas the CK group was characterized by stochastic - dominated assembly and relatively higher phylogenetic turnover. These findings highlight that LJ exerts distinct ecological forces compared to CK and SZ, reshaping microbial communities toward more phylogenetically clustered assemblages driven by deterministic processes. In the LJ group, *Metarhizium* achieved absolute dominance and complete colonization, forming a monodominant gut fungal population. In the SZ group, environmentally ubiquitous fungi—specifically *Candida parapsilosis* and *Aspergillus penicillioides*—emerged as dominant taxa, exhibiting both stable colonization in the *A. custos* gut and tolerance to SZ exposure. Both *Metarhizium* colonization and tetraniliprole (SZ) treatment induced abnormally elevated body weight growth rates in *A. custos*, alongside significant reductions in survival and suppressed activities of superoxide dismutase (SOD) and acetylcholinesterase (AchE). Notably, under *Metarhizium* stress, SOD and AchE activities displayed a dynamic trajectory—initial inhibition followed by recovery—indicating that *A. custos* has evolved adaptive defense mechanisms against *Metarhizium* toxicity. In summary, while both *Metarhizium* colonization and tetraniliprole stress exert significant adverse effects on *A. custos* physiology, these impacts are non - lethal and do not result in severe population - level mortality.

## Data Availability

The datasets presented in this study are publicly available. This data can be found here: https://www.ncbi.nlm.nih.gov/, PRJNA1321102.

## Footnotes

1
http://qiime2.org

2
https://github.com/OpenGene/fastp

3
https://ccb.jhu.edu/software/FLASH/index.shtml

4
http://www.funguild.org/

## Supplementary material

Supplementary Figure 1 Agarose gel electrophoresis of PCR products. This gel image displays the amplification results of PCR products. Lane M represents the DNA marker, with bands at 2000, 1000, 750, 500, 250, and 100 bp. Lanes 1–15 correspond to different samples. The gel shows the presence and size distribution of amplified fragments, with distinct bands indicating successful amplification for most samples. This analysis is used to assess the quality and specificity of the PCR reactions for subsequent molecular biology experiments.

Supplementary Table 1 Sequencing statistics of fungal ITS amplicons. This Supplementary Table presents the sequencing metrics of fungal ITS amplicons across different samples. The columns include: Sample (sample identifier), Seq_num (number of sequences), Base_num (total base pairs), Mean_length (mean sequence length in base pairs), Min_length (minimum sequence length in base pairs), and Max_length (maximum sequence length in base pairs). Samples are grouped as CK, LJD, LJG, SZD, and SZG, each with three biological replicates (1–3). The table also provides summary statistics for all samples, including total sequences, total base pairs, and overall mean, minimum, and maximum sequence lengths. These data reflect the sequencing depth, sequence length distribution, and quality of the fungal ITS amplicon libraries for subsequent bioinformatic analysis.

Supplementary Table 2 Data information of fungal ITS sequence processing metrics. This Supplementary Table outlines key metrics from the processing of fungal ITS sequences across all samples. Columns include sample IDs (e.g., CK-1, LJD-1), the number of raw reads, filtered reads, and final effective reads retained after quality control, as well as the corresponding retention rates (%). These data verify the efficiency of sequence filtering and ensure the reliability of high-quality reads for subsequent fungal community composition and diversity analyses.

Supplementary Table 3 Alpha diversity indices of fungal communities across samples. This Supplementary Table presents key alpha diversity indices (Shannon, Simpson, Chao1, and ACE) for fungal communities in different sample groups (CK, LJD, LJG, SZD, SZG), with three biological replicates per group. The indices reflect the richness, evenness, and overall diversity of fungal species within each sample. These data provide a basis for comparing differences in fungal community diversity among the experimental groups and supporting subsequent analyses of environmental or treatment effects on fungal assemblages.
