# Supplementary figures and images for: Risk assessment of two new pesticides based on the intestinal fungal community construction and growth status of predatory insects (Arma custos)
Source: Front Microbiol. 2025 Nov 27;16:1686765. doi: 10.3389/fmicb.2025.1686765 (PMC12695754; doi:10.3389/fmicb.2025.1686765)

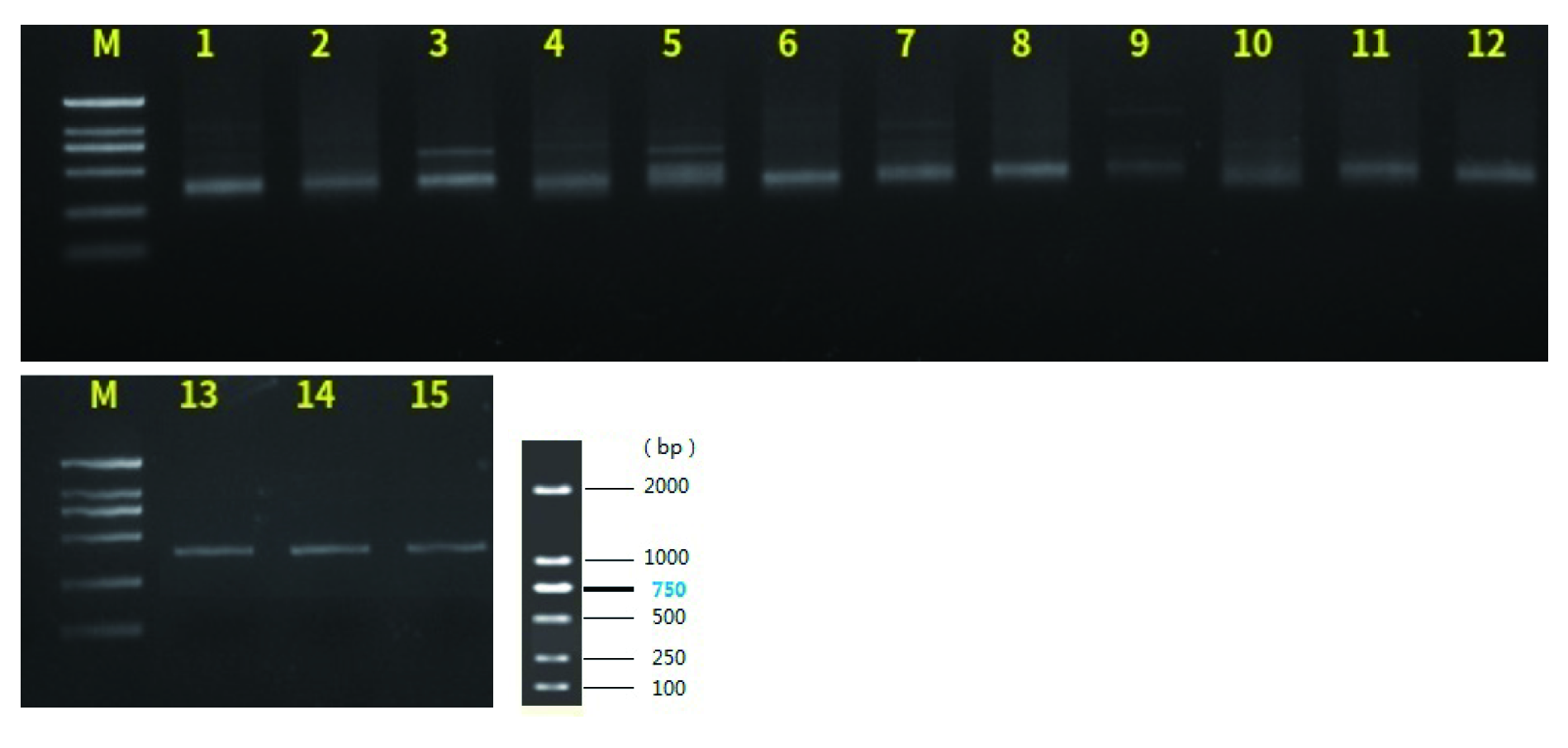

Supplement: Supplementary Figure 1 — Agarose gel electrophoresis of PCR products. This gel image displays the amplification results of PCR products. Lane M represents the DNA marker, with bands at 2000, 1000, 750, 500, 250, and 100 bp. Lanes 1–15 correspond to different samples. The gel shows the presence and size distribution of amplified fragments, with distinct bands indicating successful amplification for most samples. This analysis is used to assess the quality and specificity of the PCR reactions for subsequent molecular biology experiments. [file Image_1.tiff]
